# Fractalkine Is Expressed in Early and Advanced Atherosclerotic Lesions and Supports Monocyte Recruitment via CX3CR1

**DOI:** 10.1371/journal.pone.0043572

**Published:** 2012-08-20

**Authors:** Moritz Stolla, Jaroslav Pelisek, Marie-Luise von Brühl, Andreas Schäfer, Verena Barocke, Peter Heider, Michael Lorenz, Anca Tirniceriu, Alexander Steinhart, Johann Bauersachs, Paul F. Bray, Steffen Massberg, Christian Schulz

**Affiliations:** 1 Cardeza Foundation and the Department of Medicine, Thomas Jefferson University, Jefferson Medical College, Philadelphia, Pennsylvania, United States of America; 2 Abteilung für Gefäßchirurgie, Klinikum Rechts der Isar, Technische Universität München, Munich, Germany; 3 Deutsches Herzzentrum Medizinische Klinik, Klinikum Rechts der Isar, Technische Universität München, Munich, Germany; 4 Munich Heart Alliance, Munich, Germany; 5 Klinik für Kardiologie und Angiologie, Medizinische Hochschule Hannover, Hannover, Germany; University Medical Center Utrecht, The Netherlands

## Abstract

Fractalkine (CX3CL1, FKN) is expressed in the inflamed vascular wall and absence of FKN reduces atherogenesis. Whether FKN is expressed throughout all stages of atherosclerotic disease and whether it directly contributes to monocyte recruitment to atherosclerotic lesions is not known. We collected human atherosclerotic plaque material and blood samples from patients with carotid artery disease undergoing endarterectomy. Plaques were analyzed by immunohistochemistry and qPCR. We found that FKN is expressed at all stages of atherosclerotic lesion formation, and that the number of FKN-expressing cells positively correlates with the number of CX3CR1-positive cells in human carotid artery plaques. In the circulation, soluble FKN levels are significantly elevated in the presence of high-grade (sub-occlusive) stenosis. To determine the role of the FKN-CX3CR1 axis for monocyte adhesion in vivo we then performed intravital videofluorescence microscopy of the carotid artery in ApoE^−/−^ mice. Notably, FKN-CX3CR1 interactions are critical for recruitment of circulating monocytes to the injured atherosclerotic vascular wall. Thus, this chemokine dyad could represent an attractive target for anti-atherosclerotic strategies.

## Introduction

Rupture of carotid artery atherosclerotic plaque causes transient ischemic attack and stroke. Chemokines play a central role in atherogenesis and support the development of an unstable plaque phenotype in manifest lesions [1], [2], [3]. The membrane-bound chemokine fractalkine (CX3CL1, FKN) was detected in advanced atherosclerotic plaques [4] [5], [6]. In mouse models of atherosclerosis, absence of FKN or its receptor CX3CR1 protects from atherosclerotic lesion formation [7], [8], [9] and humans with a polymorphism for CX3CR1 exhibit a decreased risk for developing coronary artery disease [10], [11], [12]. However, it is unknown if FKN is expressed in early stages of lesion development and whether FKN directly modulates leukocyte recruitment from circulating blood in vivo. The accumulation of monocytes within the vascular wall and the subsequent differentiation into macrophages/dendritic cells (DC) plays a critical role in every stage of atherosclerotic lesion formation [13], [14]. At later stages monocyte recruitment is important for atherosclerotic plaque maintenance [15]. CX3CR1 could contribute to this process as it mediates cell migration and adhesion of monocytes, at least in vitro [16]. Experiments that followed atherosclerotic lesion development over several days suggested that CX3CR1 represents an important receptor for monocytes to accumulate in plaque in murine models of atherosclerosis [17]. However, leukocyte subsets were thought to enter the vascular wall primarily through the adventitia via vasa vasorum [18], [19]. Interestingly, recent data by Steinman and colleagues described the presence of monocyte-derived DC located in the arterial intima in healthy and atherosclerotic mouse aorta [20]. Thus, it needs to be determined whether circulating monocytes can actually bind directly to plaque and if CX3CR1 was involved in this process.

Finally, in advanced atherosclerotic lesions monocytes/macrophages contribute to plaque instability and thereby provide the foundation for atherothrombosis [21]. Monocytes promote thrombus formation by local exposition of potent procoagulant mediators such as tissue factor at the site of vascular injury [22]. However, it is not understood if and how chemokines like FKN contribute to unstable atherosclerotic disease.

In this study we addressed the presence of FKN in the vascular wall and of soluble FKN in blood at different stages of carotid artery disease in humans. Unexpectedly, we found that FKN is expressed in substantial amounts already at early stages of atherosclerosis. In ApoE-deficient (ApoE^−/−^) mice we then analyzed by real time videomonitoring acute monocyte recruitment to atherosclerotic carotids showing that FKN is capable of recruiting monocytes directly from the blood stream, and that injury of the atherosclerotic artery strongly increases FKN-CX3CR1-dependent monocyte adhesion. Our data substantiate a critical role for the FKN-CX3CR1 dyad in atherosclerosis.

## Materials and Methods

### Recruitment of Patients

A total of 137 patients with atherosclerotic carotid artery disease, who were referred to the Klinikum Rechts der Isar, Technical University Munich, for carotid endarterectomy (CEA) were studied consecutively. Patient informed and written consent was obtained in accordance with the Ethics Committee of the Faculty of Medicine of the Technical University Munich. The Ethics Committee of the Faculty of Medicine of the Technical University Munich specifically approved this study. Grade of carotid artery stenosis was determined sonographically [23].

### ELISA

Venous blood samples were taken within two days prior to surgical intervention from the cubital vein and centrifuged after clot formation to obtain serum samples. Soluble FKN were measured by ELISA (R&D Systems) as described previously [24].

### Immunohistochemistry

Carotid plaque specimens were processed as described previously [25]. Cross-sectional immunohistological analysis was performed for all carotid tissue specimens and atherosclerotic lesions were quantified according to current guidelines of the American Heart Association (AHA) [26]–[27]. Antigen retrieval was performed using heat and citrate pH 6.0 buffer. Anti-human antibodies were directed against FKN (rabbit polyclonal, TP213, Torrey Pines), CX3CR1 (rabbit polyclonal, TP502, Torrey Pines), vascular smooth muscle cells (anti-SMA, Dako, Glostrup, Denmark), endothelial cells (anti-von Willebrand factor (factor VIII), Dako) and macrophages (anti-CD68, Dako). Following primary antibody incubation, von Willebrand Factor and SMA were visualized using the APAAP ChemMate Detection Kit (rabbit anti-mouse; Dako) according to the manufacturer’s instructions. All other primary antibodies were detected by LSAB ChemMate Detection Kit (biotinylated goat anti-mouse/anti-rabbit; Dako).

For immunofluorescent staining, samples were blocked with normal goat IgG (Invitrogen) for 30 min before overnight labeling at 4°C with primary antibodies directed against human CX3CR1 (rat IgG2b, clone 2A9-1, MBL) and FKN (rabbit polyclonal, TP213, Torrey Pines). All antibodies were diluted in PBS containing 0.5% BSA and 0.3% Triton X-100. Samples were washed and incubated with Cy3-conjugated goat anti-rat (Jackson ImmunoResearch Laboratories, Inc.) and Alexa488-conjugated goat anti-rabbit secondary antibodies (Invitrogen), and 4,6-diamidino-2-phenylindole (DAPI; diluted 1∶400; Invitrogen) for nuclei counter-staining. Specimens were then prepared in mounting medium (Vector Laboratories) and visualized using a Leica TCS SP5 confocal microscope with 20×/0.5 (dry) objective. Rat IgG2b and rabbit IgG, used as negative controls, were from Abcam and Dako, respectively (Figure S1).

### Image Analysis

Analysis for FKN and CX3CR1 distribution within plaque was performed by using ImageJ software (release 1.46, National Institute of Health) and Nikon NIS Elements Version 3.0 software (NIS-Elements Advanced Research). Stained cells were labeled and positive events were determined by histogram and pixel count. Subsequently, the individual areas were encircled and the automatic pixel counting of stained cells was acquired. Analysis of FKN and CX3CR1 colocalization was performed following immunofluorescent staining as described previously [28].

### RNA Extraction from Paraffin

Quantitative analysis of FKN gene expression was performed applying a previously described method, which was optimized for formalin-fixed, paraffin-embeded tissues [29]. Total RNA was isolated using the High Pure RNA Paraffin Kit (Roche). Three 20 µm sections were cut from each tissue block, and proteinase K digestion time was 12 hours for each sample. All other steps were performed as indicated in the manufacturer’s protocol. After total RNA isolation, samples were stored at −80°C until use.

### rtPCR

Template cDNA was synthesized from 2 µg of total RNA using the High Capacity cDNA Archive Kit (Applied Biosystems). Quantitative PCR analysis was performed using SYBR Green and a GeneAmp 7700 Sequence Detection System (Applied Biosystems). The human sequences of CX3CL1 were detected by QuantiTect Primer Assays (Qiagen, Hilden, Germany) with an amplicon length of 84 bp, which is in the optimal range for PCR analysis of FFPE tissues [30]. Cycling conditions were 50°C for 2 min, 95°C for 15 min, followed by 40 cycles of denaturation at 95°C for 15 sec, and annealing and elongation at 60°C for 60 sec. Gene expression ratios for each sample (regulation factors, standard deviation) were calculated using the dCt method [31] and normalized against beta actin. Experiments were performed as triplicates, including non-Reverse Transcriptase and non-template controls. Dissociation analysis confirmed the specificity of the reaction.

**Figure 1:**
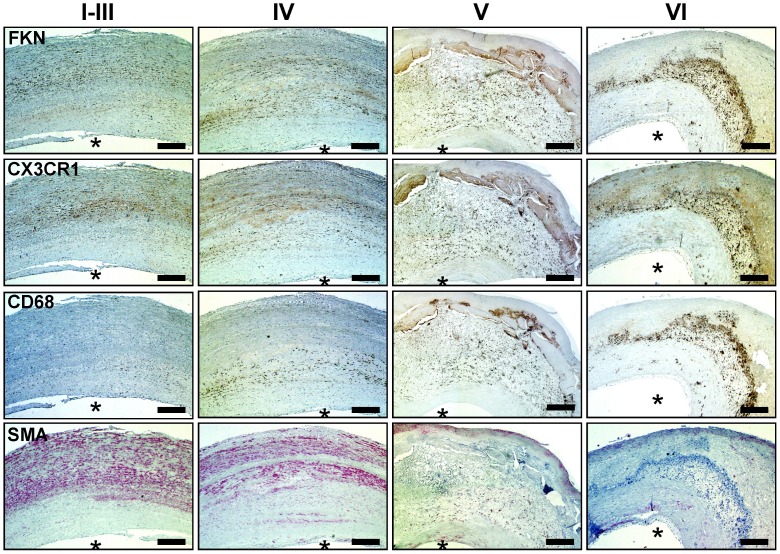
Immunohistochemistry of FKN and CX3CR1 in atherosclerotic lesions of human carotid arteries. Representative images of human atherosclerotic plaque in different stages of lesion development (I–VI). Staining was performed with antibodies against FKN, CX3CR1, the macrophage marker CD68, and α-smooth muscle actin (SMA). Bars, 200 µm. Asterisks indicate the luminal side of the vessel.

**Figure 2:**
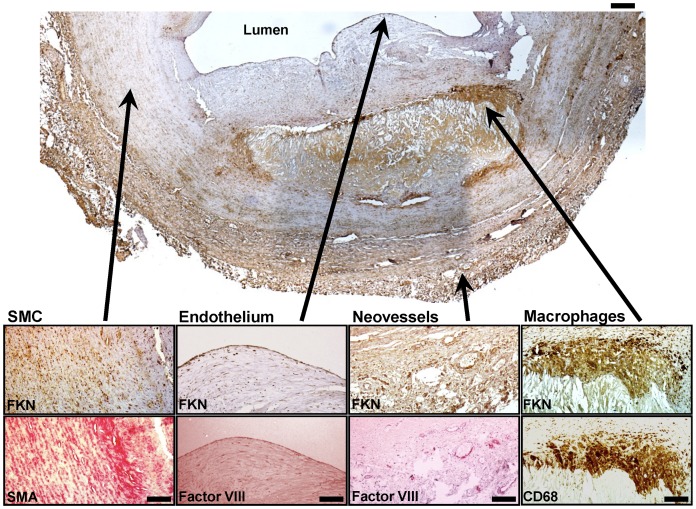
FKN positive structures in an advanced atherosclerotic lesion. Image of an advanced atherosclerotic plaque (VI) isolated by CEA from the human carotid artery. Insets magnify FKN positive structures within the plaque (upper row). Bottom row shows consecutive stainings for smooth muscle cells (SMA), endothelium and neovessels (Factor VIII), and macrophages (CD68). Upper scale bar: 200 µm, lower scale bar: 40 µm. Asterisk indicates the luminal side of the vessel.

**Figure 3:**
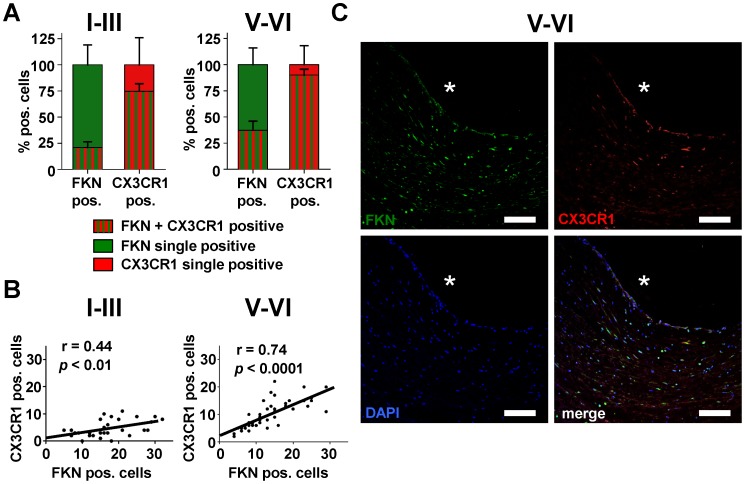
Double immunofluorescence analysis of FKN and CX3CR1 expressing cells. (A) Percent distribution of FKN (green) and CX3CR1 (red) single positive and double positive (red-green stripes) cells in early (I–III) and advanced (V–VI) plaque. n = 4 carotids per group. (B) Quantitative analysis of the number of FKN and CX3CR1 positive cells in human carotid plaque. The correlation between the co-incidence of both markers was calculated by linear regression analysis. Upper panel: early plaque (I–III), r = 0.44, p<0.01. Lower panel: advanced plaque (V–VI), r = 0.74, p<0.0001. (C) A representative section from an atherosclerotic lesion (V–VI) was stained for FKN (green), CX3CR1 (red), and DAPI (blue). Bars, 100 µm. Asterisks indicate the luminal side of the vessel.

### Cells

Monocytes were sorted from CX3CR1^GFP/+^ mice using a DAKO-Cytomation MoFlo High Speed Sorter based on expression of GFP [32]. CX3CR1-expressing lymphocyte populations were excluded using anti-mouse CD3e (clone 145-2C11), CD19 (1D3), and NK1.1 (clone PK136) antibodies (BD Pharmingen). The mouse monocyte cell line (WEHI 274.1) was purchased from European Collection of Cell Cultures (Porton Down, Salisbury, Wiltshire, UK) and maintained in suspension culture as previously described [33].

### Stable Transfection of WEHI-274.1 Cells with Sure Silencing shRNA Plasmids

Murine monocytic cells (WEHI-274.1) were transfected with 4 different short hairpin RNA (shRNA) expressing plasmids (Sure Silencing shRNA Plasmid for Mouse CX3CR1, SABiosciences) validated specifically to knock down the expression of murine CX3CR1 with a guaranteed minimized potential for non-specific and “off-target” effects. Cells were selected for neomycin resistance by G418 treatment and a pool of stably transfected cells was generated to avoid clonal selection. Among the 4 constructs with different designs for shRNA provided by the kit, one caused a significant knock down of the FKN receptor expression (Figure S2). A negative control shRNA expression vector included in the kit, which expressed a shRNA with limited homology to any known sequences in the mouse genome, was used to generate a pool of stably transfected control cells.

### Flow Cytometry

CX3CR1 surface expression was assessed by flow cytometry (Figure S2) using a rabbit anti-rat CX3CR1 antibody (rabbit polyclonal, TP501, Torrey Pines Biolabs), which is cross reactive to CX3CR1 of mice [34], and an anti-rabbit PE secondary antibody (#558416, BD Pharmingen).

### Mice

4-week-old male C57Bl6 ApoE^−/−^ (C57BL/6J-ApoE^tm1Unc^) mice (The Jackson Laboratory) were fed a 0.25% cholesterol diet (Harlan research diets, 0% cholate) for another 14 week. This atherosclerosis model leads to formation of atherosclerotic plaque in the carotid artery as published previously by Nakashima and colleagues [35] as well as by our group [36], [37]. Experiments were performed using 18-week-old male animals. CX3CR1^GFP/GFP^ (B6.129P-CX3CR1^tm1Litt^/J) mice (The Jackson Laboratory) express the green fluorescence protein (GFP) under the control of the endogenous CX3CR1 promoter [38]. Heterozygous CX3CR1^GFP/+^ mice were used for experiments. All mice were maintained in a pathogen-free environment. All experimental procedures on animals met the requirements of the German legislation on protection of animals and were approved by the Government of Bavaria, Germany.

**Figure 4:**
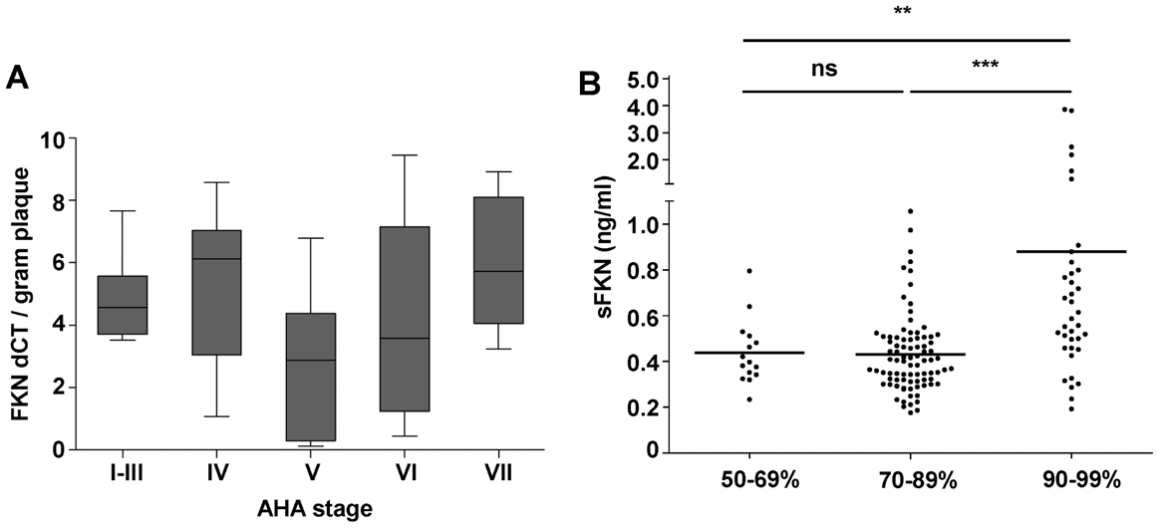
Quantitative analysis of FKN expression and FKN serum levels. (A) PCR analysis of FKN expression in atherosclerotic plaques of different AHA stages. Graph indicates relative expression ratio as calculated from the real-time PCR efficiencies and the crossing point deviation of FKN versus control. Mean FKN expression per gram plaque tissue did not differ significantly between AHA stages I–VI. (B) Serum levels of sFKN in 10 patients with moderate (50–69%), 83 with advanced (70–89%), and 44 with subocclusive (90–99%) carotid artery stenosis. Patients with subocclusive stenosis showed significantly higher levels of sFKN. **p<0.001 for 50–69% vs. 90–99% (0.44±0.04 ng/ml vs. 0.88±0.14 ng/ml). ***p<0.0001 for 70–89% vs. 90–99% (0.43±0.02 ng/ml vs. 0.88±0.14 ng/ml). p = ns for 50–69% vs. 70–89%.

### Intravital Microscopy Animal Model

Intravital microscopic studies of murine carotid arteries were performed as previously described [37], [39]. The right jugular vein of anesthetized ApoE^−/−^ mice was isolated and a catheter was inserted. 5×10^4^ murine monocytes or 5×10^6^ WEHI-274.1 cells were injected intravenously via the jugular catheter. Endogenous expression of GFP was sufficient for intravital microscopy of monocytes from CX3CR1^GFP/+^ mice, while WEHI-274.1 were labeled with 5-carboxyfluorescein diacetate succinimidyl ester (DCF). Interaction of monocytes or WEHI cells with the vascular wall of atherosclerotic carotids was visualized using a BX51W1 microscope (20× water immersion objective, Olympus) equipped with a MT20 monochromator for epi-illumination as reported previously [37], [39]. Therefore, the common carotid artery was carefully exposed at a distance of 3 mm distal and 7 mm proximal of the carotid bifurcation. Monocyte interaction with the vessel wall was monitored over 30 minutes, then ligature injury was induced and imaging was continued for another 30 minutes. To induce carotid injury, the vessel was ligated vigorously for 5 min, resulting in a circular incision into the endothelium and exposure of the subendothelial collagen layer [40]. The ligation was removed after 5 min. Injury of the vessel wall leads to recruitment of circulating blood cells to the site of injury endothelial denudation [41]. Number of adherent cells was quantified and given as n/mm^2^ vessel area (n = 4–8 carotid arteries per group). To study the role of FKN for monocyte recruitment *in vivo*, we pre-treated mice with either a function blocking anti-mouse FKN antibody (rabbit polyclonal, TP233, Torrey Pines) or rabbit IgG. TP233 or control IgG were pre-injected 24 hrs (4 mg/kg intraperitoneal) and 15 min (2 mg/kg intravenous) prior to experiments [42].

### Statistical Analysis

Data represent mean ± SEM. Kolmogorov–Smirnov test was used to assess the normal distribution of the experimental data. In case of normal distribution the unpaired, 2-tailed t-test was applied. For all other data the non-parametric Mann-Whitney U-test was used. A value of P<0.05 was regarded significant. The correlation between FKN and CX3CR1 positive cells was performed using linear regression analysis (Prism 5 Software, Graphpad).

**Figure 5:**
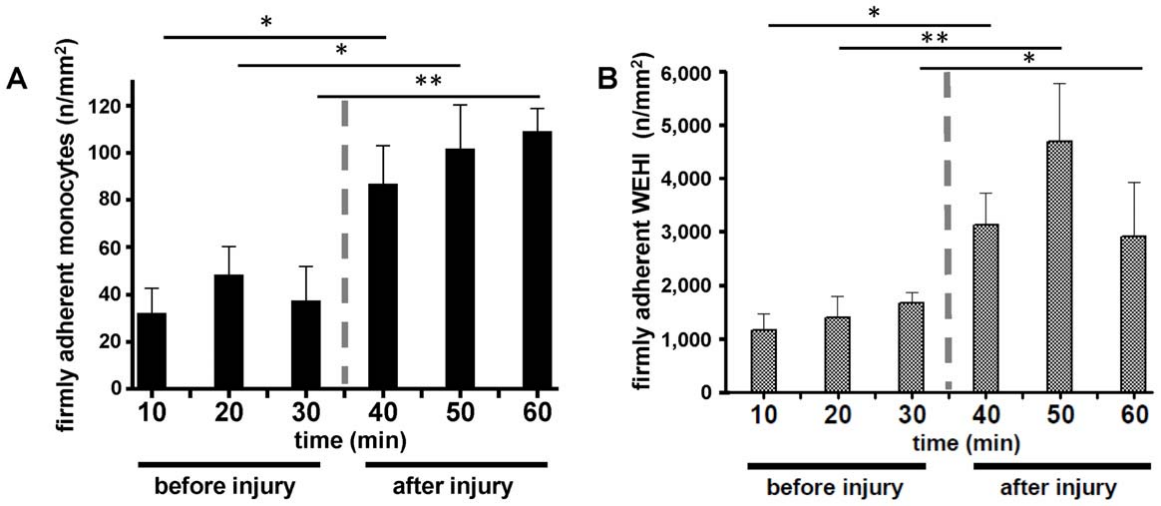
Monocyte and WEHI 274.1 recruitment to intact and injured atherosclerotic carotid artery lesions. (**A**) Monocyte adhesion to atherosclerotic endothelium (indicated ‘before injury’) and to the injured atherosclerotic vascular wall (indicated ‘after injury’) in ApoE^−/−^ mice (**B**) WEHI 274.1 adhesion to inflamed atherosclerotic endothelium and to the injured atherosclerotic vascular wall in ApoE^−/−^ mice. Adhesion of monocytes and WEHI 274.1 was significantly increased following injury. n = 4–8, *p<0.05, **p<0.001.

**Figure 6:**
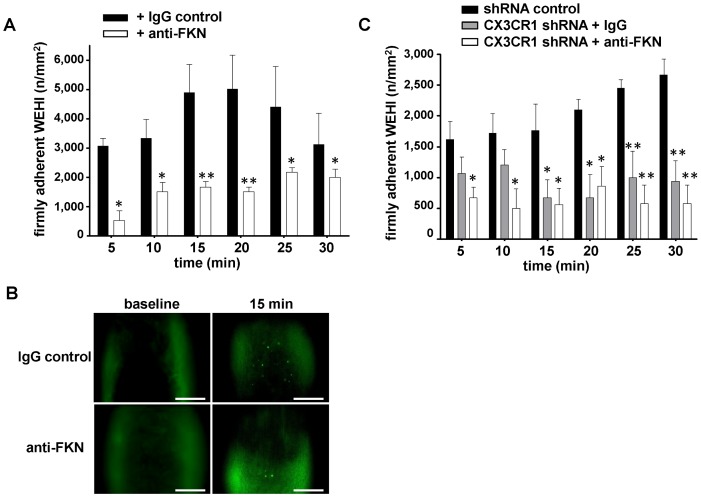
FKN and CX3CR1 mediate WEHI 274.1 recruitment to injured atherosclerotic carotid arteries. (**A**) WEHI 274.1 adhesion to mechanically injured atherosclerotic carotids was studied over 30 minutes in the presence of an IgG control antibody (black bars) or a function blocking anti-FKN antibody (white bars). (**B**) Representative intravital microscopic images from mouse carotid arteries at baseline (left) and 15 minutes (right) after injury of the atherosclerotic vascular wall, pretreated with either an anti-FKN antibody (upper row) or an isotype IgG control antibody (lower row). WEHI 274.1 cells were stained with DCF (green). Bars, 50 µm. n = 4–8, *p<0.05, **p<0.01. (**C**) WEHI 274.1 adhesion is dependent on FKN-CX3CR1 interactions. WEHI 274.1 were transfected with shRNA-encoding plasmids to silence CX3CR1 expression (grey and white bars) or transfected with a plasmid encoding a scrambled control shRNA (black bars). Prior to transfusion the animals were pretreated either with a rabbit IgG control antibody (grey bars) or a function blocking anti-FKN antibody (white bars). WEHI 274.1 adhesion to injured atherosclerotic carotids was analyzed over 30 minutes.

## Results

To systematically analyze FKN expression in atherosclerotic plaque of human carotid arteries we obtained tissue specimen from patients undergoing CEA. Unexpectedly, we detected FKN at all stages of atherosclerotic lesion development using qPCR analysis and immunohistochemistry. FKN positive staining was found in various areas of the vascular wall (Figure 1) and comprised macrophages, smooth muscle cells and endothelium, including intraplaque microvessels (Figure 1, 2, S3, S4). In both early and advanced stages of lesion development, FKN was more prominent in deeper layers of the vessel wall (Figure 1, S5). In advanced plaques (V–VI), we also found relative enrichment in the plaque core region while FKN staining was less prominent in the overlying fibrous cap (Figure 1, 2, S5). Importantly, staining for the FKN receptor CX3CR1 closely collocated with FKN-positive structures (Figure 1, 3). In fact, most CX3CR1 positive events were also positive for FKN and the percentage of double-positive cells increased from early to advanced stages (Figure 3A). Further, we determined a positive correlation between the number of FKN-positive and CX3CR1-positive events, which was more significant in advanced lesions (Figure 3B). The latter finding is comparable to previous reports by Lucas and colleagues on patients with coronary artery atherosclerosis [28].

To quantify FKN expression within plaque we next performed PCR analysis of plaque specimens. In line with the findings obtained by immunohistochemistry, FKN was expressed throughout all stages of atherosclerotic disease (Figure 4A). High amounts of FKN were expressed already at early stages of lesion development. However, mean amounts of CX3CL1 per gram plaque tissue were not statistically different in-between groups (Figure 4A). On inflamed endothelium and within the vascular wall, FKN is expressed as a transmembranous molecule. However, the chemokine domain can be cleaved from the cell surface to produce a soluble chemoattractant [43]. Elevated levels of soluble FKN (sCX3CL1, sFKN) have been described in chronic inflammatory disease in humans and in patients with acute coronary syndrome [5]. Thus, we next assessed whether levels of circulating sFKN are associated with the degree of carotid artery stenosis. Patients with moderate (50–70% stenosis) and advanced (70–90% stenosis) carotid artery disease showed similar levels of circulating sFKN. However, patients with carotid artery stenosis near occlusion (90–99% stenosis) showed significantly elevated levels of sFKN (Figure 4B). Importantly, patient characteristics were well balanced between the groups and did not differ significantly in terms of sex, age, risk factors and medication (Table S1).

FKN and its receptor CX3CR1 play an important role in atheroprogression [7], [8], [9]. *In vitro*, membrane-bound FKN mediates adhesion of leukocyte subsets [16], [44]. However, whether the FKN-CX3CR1 axis is involved in the direct recruitment of circulating monocytes to the atherosclerotic vascular wall *in vivo* is not known. To address this question, we first isolated murine monocytes from CX3CR1^GFP/+^ mice and injected 5×10^4^ cells intravenously into 18-week-old ApoE^−/−^ mice. We then studied monocyte adhesion at the carotid artery by intravital microscopy. Importantly, we directly observed the recruitment of monocytes to atherosclerotic endothelium, although total events were relatively low within 1 hour of microscopy (Figure 5A). However, when we mechanically injured the atherosclerotic artery, a significant increase in adherent CX3CR1^GFP/+^ monocytes was observed (Figure 5A). As FKN is relevantly expressed in atherosclerotic arteries, the findings provoked us to further analyze the role of FKN in the process of monocyte recruitment to atherosclerotic lesions. To increase the number of adhesion events we next used cultured murine monocytes (WEHI-274.1) and injected 5×10^6^ cells into ApoE^−/−^ mice. As observed with GFP+ monocytes (Figure 5A), adhesion of WEHI cells also increased strongly following injury (Figure 5B). Importantly, pretreatment with a neutralizing antibody diminished WEHI recruitment (Figure 6A,B). Thus, FKN expressed within atherosclerotic lesions is an important mediator for the recruitment of circulating blood monocytes.

Whether FKN mediates monocyte adhesion directly via CX3CR1 is not known. Early work suggested that FKN interactions with CX3CR1 should be strong enough to mediate firm adhesion under conditions of high wall shear stress [45]. To test whether FKN and CX3CR1 are both involved in monocyte recruitment in vivo, we next injected WEHI cells into ApoE^−/−^ after silencing of CX3CR1. shRNA-treatment significantly reduced WEHI-247.1 recruitment to the injured carotid artery. Unexpectedly, additional pre-incubation with FKN antibody did not further affect the adhesion of shRNA-treated WEHI-274.1 cells (Figure 6C), suggesting that the reduction observed was mediated primarily by FKN-CX3CR1 interaction. Thus, our findings suggest a critical role of the FKN-CX3CR1 axis for monocyte recruitment to the injured vessel wall in this mouse model of accelerated atherosclerosis.

## Discussion

FKN plays an important role in atherosclerotic vascular disease. Although some progress has been made in recent years to better understand the role of this chemokine in atherosclerosis and the mechanisms underlying monocyte recruitment, some issues remained elusive. For example, it was unknown at which stage of disease FKN becomes expressed and whether levels of circulating sFKN may reflect increased FKN expression within the vascular wall. While FKN is not detectable in the vessel wall of healthy mice [46] and human subjects [6], it has been described in advanced atherosclerotic lesions in different vascular beds [4]. Using immunohistochemistry and real-time PCR we show here that FKN is substantially expressed already at very early stages of atherosclerosis. This suggests a role of this chemokine in the initial stages of lesion development in addition to its relevance in advanced disease. Interestingly, FKN positive staining was detected among others on endothelium of atherosclerotic carotid arteries. This finding was not observed in previous studies on human atherosclerosis [6], [28], but it is in line with FKN expression analysis in atherosclerotic endothelium of *ApoE* ^−/−^ mice [8].

Leukocytes play a critical role in atherosclerosis. While leukocyte subsets reside within the normal vascular wall, their number strongly increases with the onset of atherogenesis [19]. In addition to constitutive leukocyte surveillance and trafficking, monocytes and lymphocytes are then specifically recruited in larger amounts to the site of lesion formation. This process is controlled by distinct signals that mediate their migration and adhesion. It involves eg. L-selectin and CXCR6, the only known receptor to the membrane-bound chemokine CXCL16 [18]. There is also evidence pointing to a role of CX3CR1 for monocyte recruitment into plaque [17]. Membrane-bound FKN mediates migration of immune cells and smooth muscle cells during atherogenesis [9], [28]. In line with these studies, we found that the number of CX3CR1-positive cells in carotid artery lesions increased in the course of atherogenesis and correlated significantly with the presence of FKN-positive structures. However, monocytes are thought to accumulate within the vascular wall entering primarily through the vasa vasorum located in the adventitia [18], [19]. Using intravital microscopy we show here that blood-borne monocytes can also be directly recruited to the luminal side of the vessel wall. We found that both isolated murine monocytes as well as cultured monocytes (WEHI 274.1) adhered to atherosclerotic endothelium and injury of carotid artery plaque strongly increased monocyte adhesion. This process was mediated predominantly by FKN and pretreatment with a neutralizing anti-FKN antibody diminished monocyte recruitment. Our findings not only highlight the pathophysiological relevance of FKN in CAD, but we here identified a mechanism by which monocyte-dependent macrophages/DC might accumulate within the arterial intima.

Initial work on FKN suggested that its interactions with CX3CR1 are strong enough to mediate firm adhesion under conditions of high wall shear stress [45]. However, flow experiments *in vitro* indicated that FKN only effectively mediated leukocyte adhesion in the presence of another adhesion molecule, like VCAM-1 or P-selectin, that supports capture and rolling before FKN is capable of mediating firm adhesion [44], [47], [48]. As determined in the present study FKN-mediated monocyte binding *in vivo* involves predominantly its receptor CX3CR1. Thus, this interaction seems sufficient for monocyte recruitment *in vivo*. A possible explanation for this finding is that the cumulative density of FKN within advanced atherosclerotic lesions is higher than on inflamed endothelium *in vitro*. However, involvement of other adhesion molecules cannot be excluded in this model.

It is tempting to speculate that by recruiting monocytes to the injured atherosclerotic plaque FKN-CX3CR1 might contribute to plaque vulnerability and rupture, which may lead to atherothrombosis and stroke as one major clinical sequela. Indeed, locally recruited monocytes foster arterial thrombosis for example by release of tissue factor-bearing microparticles [22], [49].

In addition to its’ existence as a membrane-bound molecule, soluble FKN also seems to be of pathophysiological relevance. Membrane-bound FKN is cleaved by metallopeptidases, eg. ADAM 10 and ADAM 17 [43], and serves as a chemoattractant for different leukocyte subsets [13]. Circulating sFKN can be detected in blood of patients with various inflammatory diseases as well as healthy subjects [5], [13], [50]. We found that sFKN blood levels are similar in patients with moderate and advanced carotid artery stenosis. Interestingly, values are well comparable to sFKN levels in patients with stable coronary artery disease [5]. However, severe stenosis leading to subtotal occlusion of the carotid artery significantly increased sFKN levels in circulating blood. In contrast, expression of the chemokine within the vascular wall can already be detected at early stages of carotid artery disease. Given our findings and the previous data on patients with coronary artery disease we believe that sFKN is not suitable as a biomarker for patients with atherosclerosis per se, but might be useful to identify patients with unstable disease.

In conclusion, our findings suggest that FKN and CX3CR1 could represent an interesting therapeutic target in patients with atherosclerotic vascular disease. This seems especially relevant in the context of CX3CR1 antagonists being engineered for treatment in humans [51].

## Supporting Information

Figure S1 **Control staining of atherosclerotic plaque.**
(TIF)

Figure S2 **Flow cytometry of transfected WEHI 274.1 cells.**
(TIF)

Figure S3 **FKN positive structures in an advanced atherosclerotic lesion.**
(TIF)

Figure S4 **Microvessels in atherosclerotic plaque.**
(TIF)

Figure S5 **Distribution of FKN and CX3CR1 in the atherosclerotic vascular wall.**
(TIF)

Table S1 **Patient characteristics.**
(DOC)
