# Supplementary figures and images for: Fractalkine Is Expressed in Early and Advanced Atherosclerotic Lesions and Supports Monocyte Recruitment via CX3CR1
Source: PLoS One. 2012 Aug 20;7(8):e43572. doi: 10.1371/journal.pone.0043572 (PMC3423360; doi:10.1371/journal.pone.0043572)

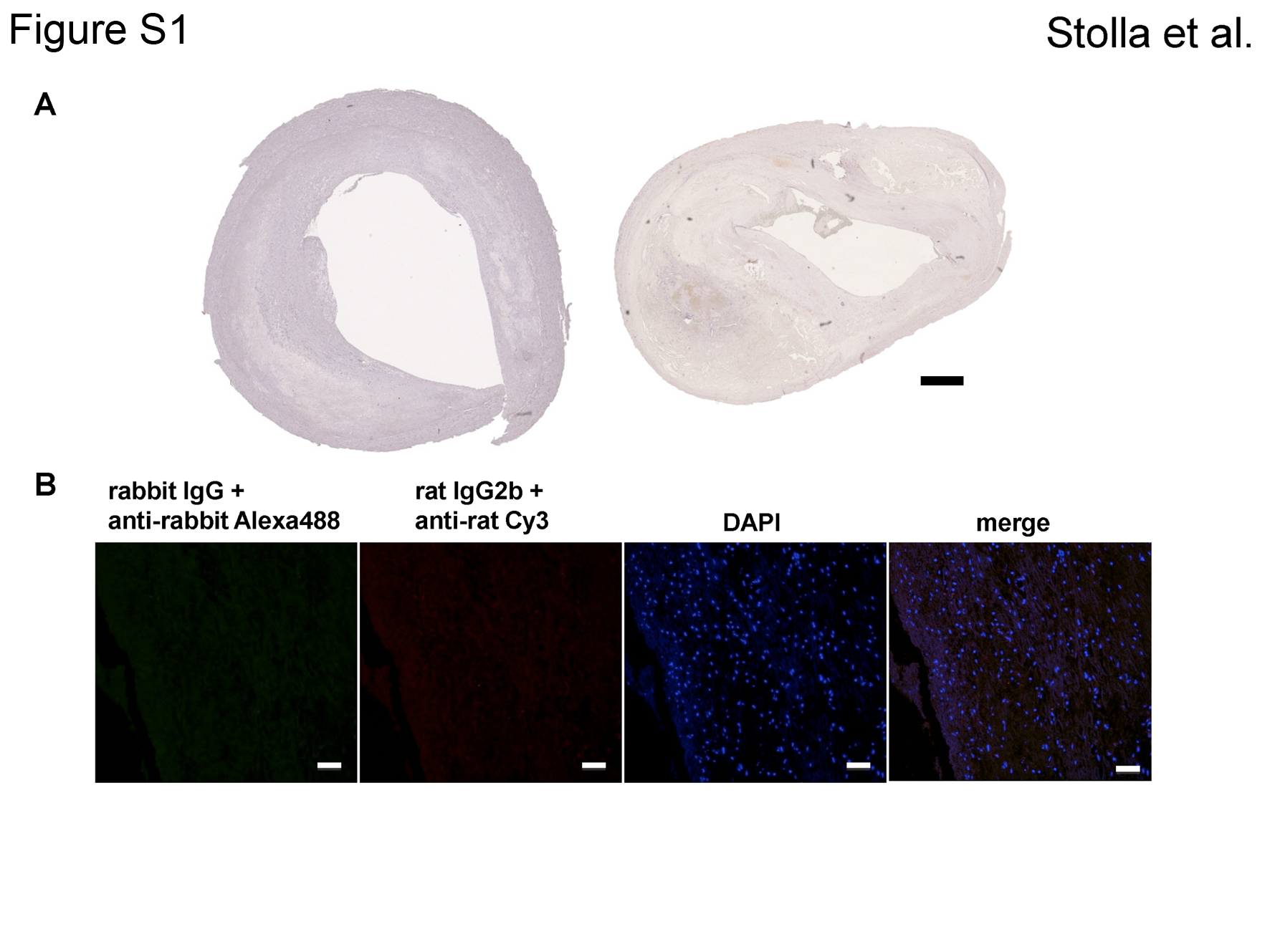

Supplement: Figure S1 — Control staining of atherosclerotic plaque. (TIF) [file pone.0043572.s001.tif]

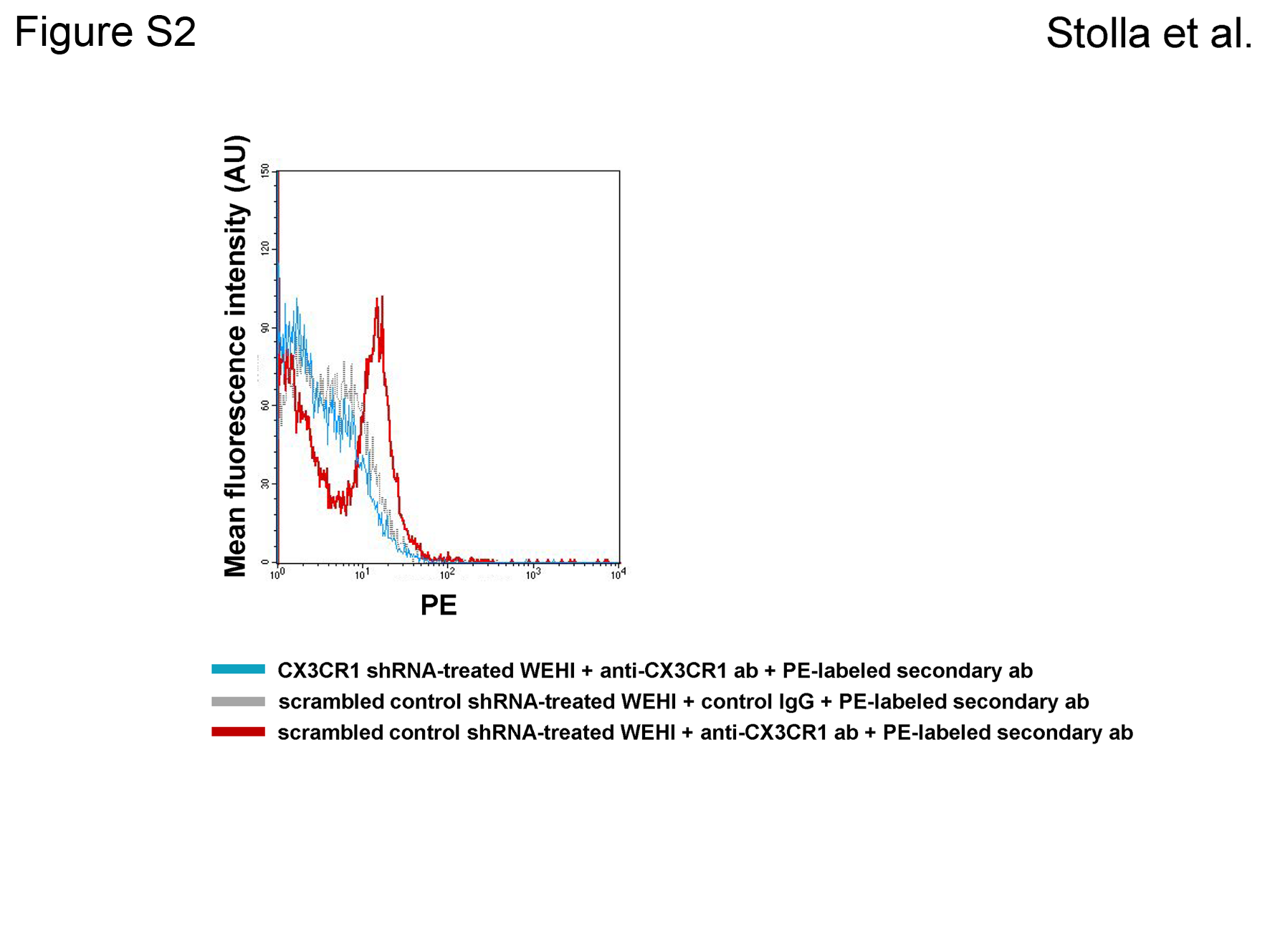

Supplement: Figure S2 — Flow cytometry of transfected WEHI 274.1 cells. (TIF) [file pone.0043572.s002.tif]

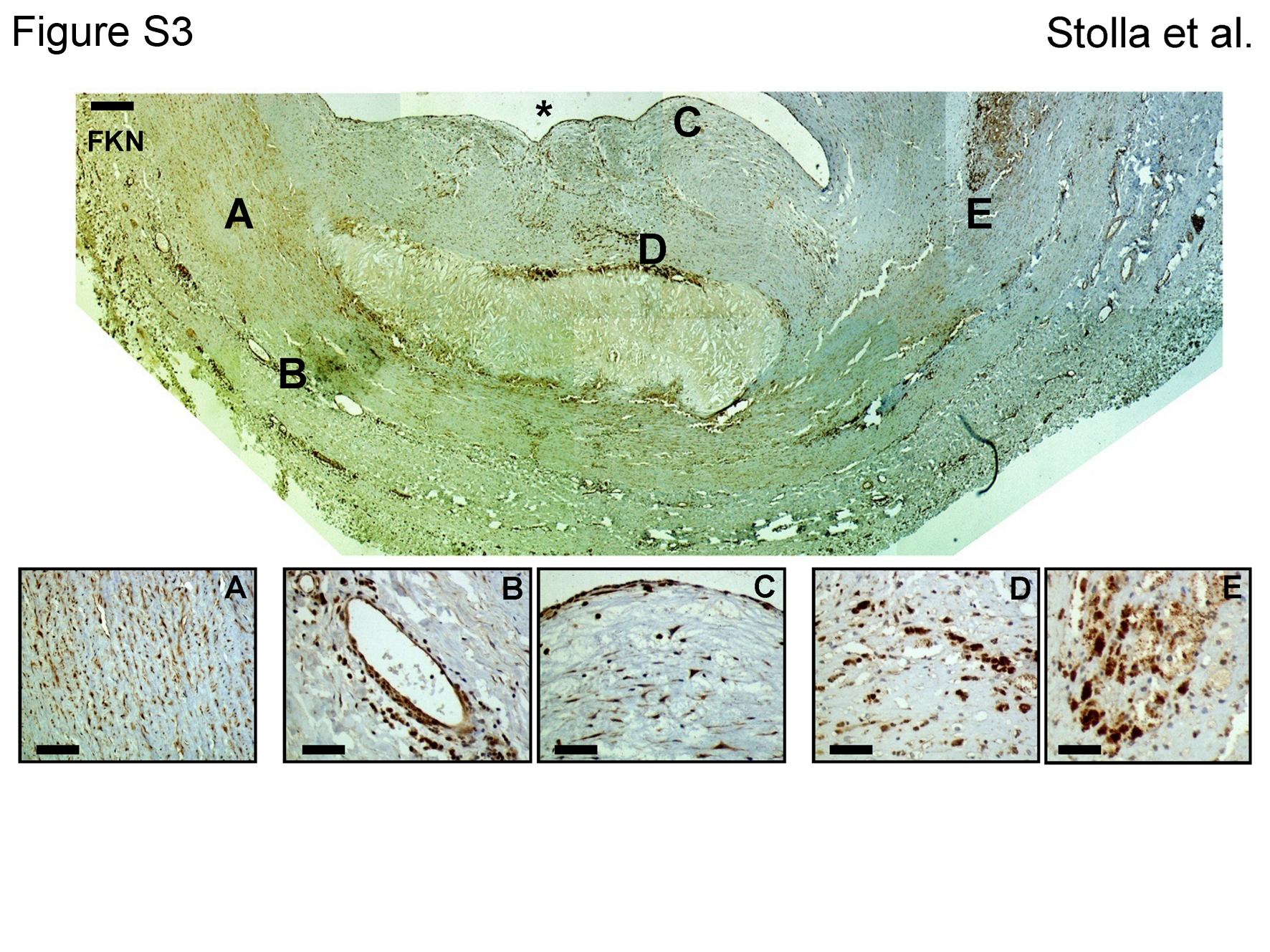

Supplement: Figure S3 — FKN positive structures in an advanced atherosclerotic lesion. (TIF) [file pone.0043572.s003.tif]

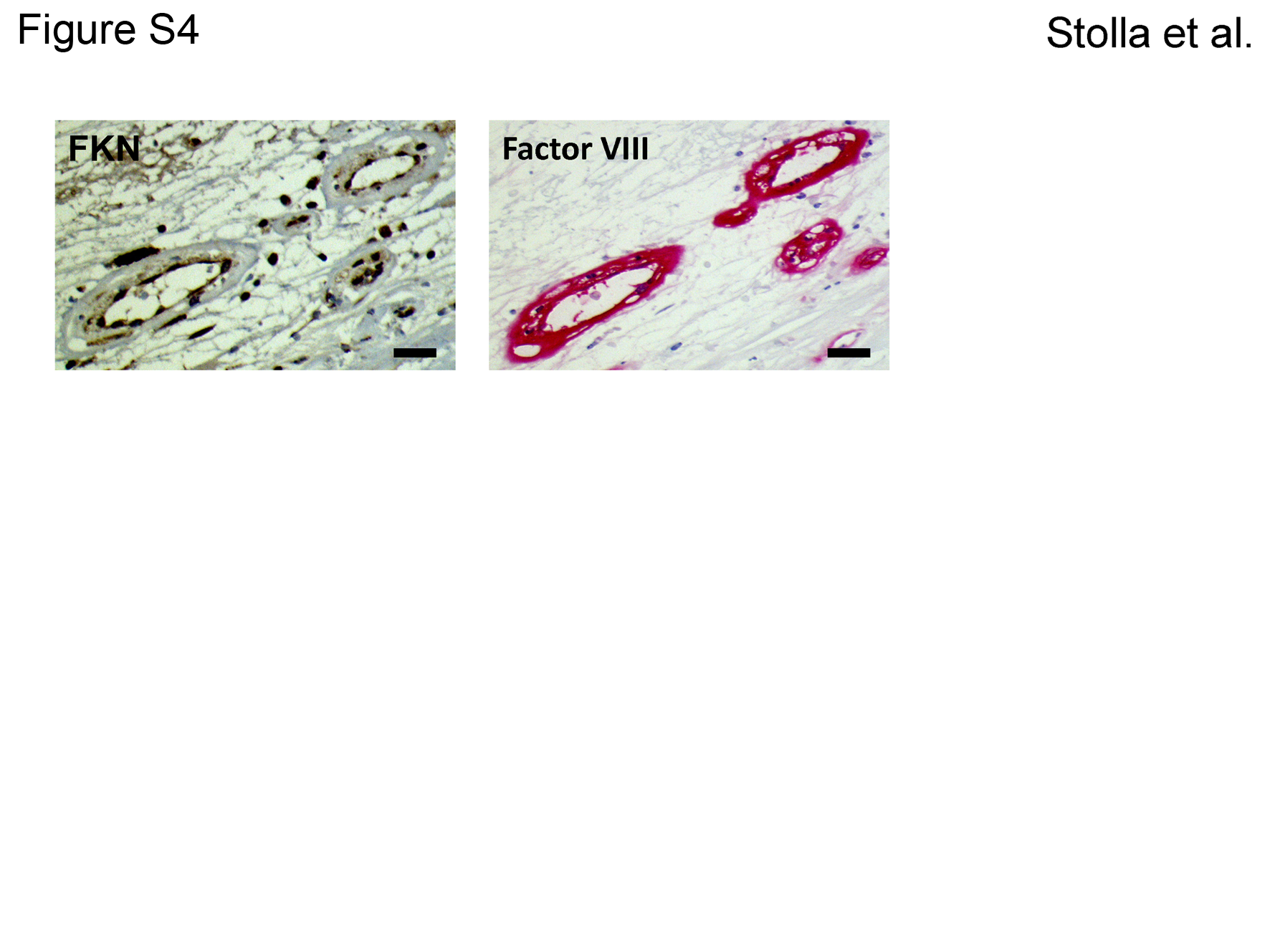

Supplement: Figure S4 — Microvessels in atherosclerotic plaque. (TIF) [file pone.0043572.s004.tif]

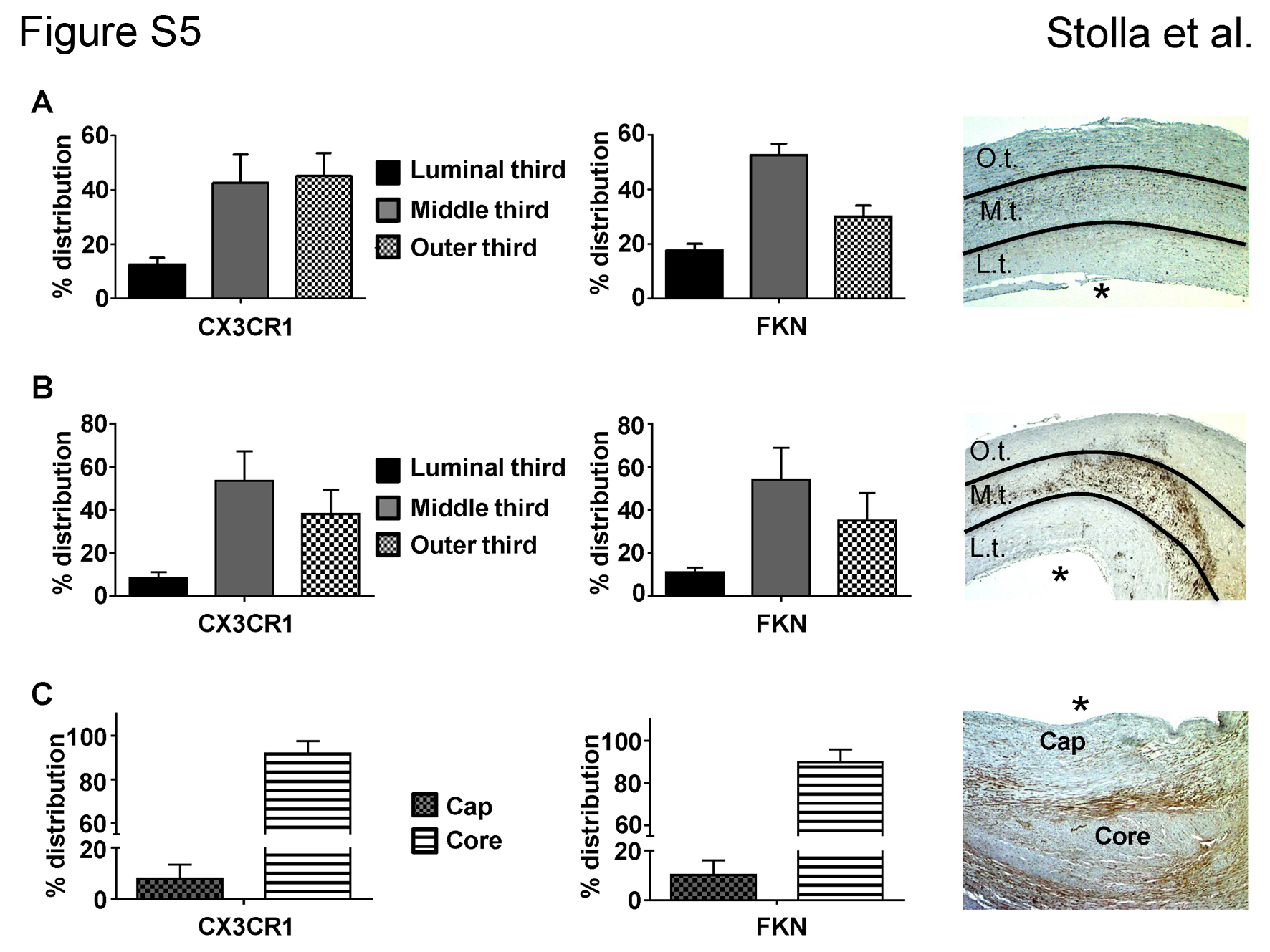

Supplement: Figure S5 — Distribution of FKN and CX3CR1 in the atherosclerotic vascular wall. (TIF) [file pone.0043572.s005.tif]
