# Supplementary material for: Fractalkine Is Expressed in Early and Advanced Atherosclerotic Lesions and Supports Monocyte Recruitment via CX3CR1
Source: PLoS One. 2012 Aug 20;7(8):e43572. doi: 10.1371/journal.pone.0043572 (PMC3423360; doi:10.1371/journal.pone.0043572)
Supplement: Table S1 — Patient characteristics. (DOC) [file pone.0043572.s006.doc]

**Table S1: Patient characteristics**

| Degree of ACI stenosis | **50-69%** | **70-89%** | **90-99%** | **p-value** |
| --- | --- | --- | --- | --- |
| **N** | 10 | 83 | 44 |  |
| Sex (m/f, m[%]) | 8/2[80] | 56/27[68] | 25/19[57] | 0.29 |
| Age (years) | 69±9 | 69±8 | 69±9 | 0.92 |
| **Risk factors** | | | | |
| Diabetes mellitus (%) | 20 | 24 | 27 | 0.87 |
| Hyperlipoproteinemia (%) | 60 | 74 | 70 | 0.67 |
| Hypertension (%) | 90 | 84 | 97 | 0.12 |
| Smoker (%) | 40 | 50 | 41 | 0.6 |
| **Medication** | | | | |
| ASA/Clopidogrel (%) | 100 | 99 | 100 | 0.71 |
| Statins (%) | 60 | 64 | 58 | 0.84 |
| **Coronary artery disease (%)** | 50 | 34 | 30 | 0.49 |
